# Public Health Adaptation to Climate Change in OECD Countries

**DOI:** 10.3390/ijerph13090889

**Published:** 2016-09-07

**Authors:** Stephanie E. Austin, Robbert Biesbroek, Lea Berrang-Ford, James D. Ford, Stephen Parker, Manon D. Fleury

**Affiliations:** 1 Department of Geography, McGill University, Burnside Hall Building Room 705, 805 Sherbrooke Street West, Montreal, QC H3A 0B9, Canada; lea.berrangford@mcgill.ca (L.B.-F.); james.ford@mcgill.ca (J.D.F.); 2 Tracking Adaptation to Climate Change Collaboration (TRAC3), McGill University, Burnside Hall Building Room 705, 805 Sherbrooke Street West, Montreal, QC H3A 0B9, Canada; 3 Public Administration and Policy Group, Wageningen University and Research Centre, P.O. Box 8130, 6700EW Wageningen, The Netherlands; robbert.biesbroek@wur.nl; 4 Enteric Surveillance and Population Studies Division, Centre for Food-Borne, Environmental and Zoonotic Infectious Diseases, Public Health Agency of Canada, 255 Woodlawn Road West, Unit 120, Guelph, ON N1H 8J1, Canada; stephen.parker@phac-aspc.gc.ca (S.P.); Manon.D.Fleury@phac-aspc.gc.ca (M.D.F.)

**Keywords:** climate change, adaptation, public health, OECD countries, adaptation tracking

## Abstract

Climate change is a major challenge facing public health. National governments play a key role in public health adaptation to climate change, but there are competing views on what responsibilities and obligations this will—or should—include in different nations. This study aims to: (1) examine how national-level public health adaptation is occurring in Organization for Economic Cooperation and Development (OECD) countries; (2) examine the roles national governments are taking in public health adaptation; and (3) critically appraise three key governance dimensions of national-level health adaptation—cross-sectoral collaboration, vertical coordination and national health adaptation planning—and identify practical examples suited to different contexts. We systematically reviewed publicly available public health adaptation to climate change documents and webpages by national governments in ten OECD countries using systematic web searches, assessment of self-reporting, and content analysis. Our findings suggest national governments are primarily addressing infectious disease and heat-related risks posed by climate change, typically emphasizing capacity building or information-based groundwork initiatives. We find national governments are taking a variety of approaches to public health adaptation to climate change that do not follow expected convergence and divergence by governance structure. We discuss practical options for incorporating cross-sectoral collaboration, vertical coordination and national health adaptation planning into a variety of contexts and identify leaders national governments can look to to inform their public health adaptation planning. Following the adoption of the Paris Agreement and subsequent increased momentum for adaptation, research tracking adaptation is needed to define what health adaptation looks like in practice, reveal insights that can be taken up across states and sectors, and ensure policy orientated learning.

## 1. Introduction

The health impacts of climate change are expected to be significant, and future climate change is projected to further affect the magnitude and frequency of health risks, including morbidity and mortality due to extreme weather, increased respiratory illness, and changing prevalence, incidence, and distribution of infectious diseases [1]. Indirect impacts are also expected, and include compromised food security, exacerbation of mental health problems, and a magnification of social gradients in health (the stratification of health outcomes by socioeconomic status [2,3]) [4,5]. Climate change has been identified as one of the greatest threats to health globally this century, and leading medical associations and the World Health Organization have called for increased action to prepare for impacts [6,7,8,9,10,11].

Given recognition of the risks posed by climate change, adaptation has emerged as a key component of climate policy in a health context [12]. In this paper adaptation is referred to as “the process of adjustment to actual or expected climate and its effects” (p. 1758) [13]. In the health context adaptation is synonymous with prevention [14], where primary prevention aims to reduce exposure to risks, secondary prevention aims to prevent the onset of adverse health outcomes, and tertiary prevention aims to reduce morbidity and minimize impacts [14,15,16].

The Paris Agreement, adopted by the member states of the United Nations Framework Convention on Climate Change (UNFCCC) in December 2015, places adaptation firmly on the policy agenda, and, for the first time in the history of the UNFCCC process, on the same level as mitigation [17]. As national governments begin or continue to consider their health adaptation options, research tracking adaptation is needed to examine if and how adaptation is occurring, define what health adaptation looks like in practice, identify leaders to look for best practices and innovative solutions, reveal insights that can be applied across states and sectors, and monitor changes in adaptation over time [17,18,19,20]. Massey et al. found that European countries frequently model their adaptation policies, practices, and institutions on other countries’ experiences. These findings suggest that research reviewing, comparing, and contrasting national adaptation initiatives may be fruitful for national governments embarking upon adaptation [21]. Taking stock of and understanding the patterns of how public health adaptation takes place across countries and the influence of key governance characteristics is crucial to ensure policy orientated learning and transfer lessons learned and best practices on public health adaptation across states.

Many national governments have started to recognize the impacts of climate change on health in their national adaptation strategies [22]. Countries with diverse state structures are expected to adapt differently to the impacts of climate change because their roles and responsibilities are distributed differently [23]. This means that some states will have adopted legislation to push for adaptation and/or created new institutional arrangements to ensure coherence and consistency in implementing public health adaptation across scales and across multiple policy domains [24,25]. Adopting legislation and ensuring interdepartmental coordination are reported in some contexts as crucial conditions for successful adaptation [25,26,27]. Other states, however, do not take a centralized authoritative or coordinating role beyond assigning responsibility for public health adaptation to specific authorities and institutions through a form of self-governance [28,29]. 

In this study, we systematically review national-level public health adaptation to climate change in ten Organization for Economic Cooperation and Development (OECD) countries. We aim to: (1) examine how national-level public health adaptation is occurring in OECD countries in terms of health risks addressed, types of adaptation initiatives being planned or implemented, incorporation of inter-sectoral activities, and state of national health adaptation planning; (2) examine the roles national governments are taking in public health adaptation; and (3) critically appraise three key governance dimensions of national-level health adaptation—cross-sectoral collaboration, vertical coordination and national health adaptation planning—and identify practical examples suited to different contexts.

Few studies have systematically tracked public health adaptation at the national-level in developed countries, exceptions being Panic and Ford who examined national adaptation to infectious disease risks in OECD countries, and Lesnikowski et al. who quantitatively assessed national health adaptation among high income countries [24,30]. However, both studies primarily presented generalized trends across sampled countries, rather than analyzing how health adaptation is being governed across countries. This study attempts to respond to this gap to achieve *breadth* in understanding the current adaptation landscape for public health through a systematic comparative analysis of adaptation across countries, but also *depth* through our qualitative examination of national-level public health adaptation to climate change.

We first contextualize this study (Section 2), reviewing academic literature on national governments’ role in adaptation, approaches to public health adaptation, and transferable dimensions of public health adaptation policy. Following our empirical methods to systematically review national-level public health adaptation (Section 3), we describe the current state of reported national-level public health adaptation (Section 4). We discuss the roles that national governments have taken for public health adaptation, identify options for cross-sectoral collaboration, vertical coordination and national adaptation planning for public health, and the limitations of this study (Section 5). We conclude by arguing that reviewing national-level public health adaptation to climate change provides a learning opportunity for other governments, and that, while adaptation decisions are context dependent, the three key governance dimensions identified are broadly relevant—though manifest differently through local contexts—across governing structures and traditions.

### Role of National Governments in Public Health Adaptation

The assumption “mitigation is global, adaptation is local” has dominated adaptation discourse since the signing of the UNFCCC in 1992, but is increasingly questioned [31,32,33] as empirical studies find that there is a lack of adaptive capacity (e.g., financial resources, technology, and training) at the local level, and local stakeholders require greater support, steering, and coordination from higher levels of government to adapt [26,27,33,34,35,36]. Moreover, adaptation requires integration in existing policy and governing structures to be most effective. National governments undeniably play a key role in health adaptation as they have profound (legal and resource) capacities to steer adaptation in general and public health adaptation in particular, however precisely what that role is—or should be—and its magnitude relative to other contexts, has been debated in academic literature [26,36]. Amundsen et al. and Paterson et al., for example, argue that the national level’s role is to place adaptation on the policy agenda and signal its importance to sub-national governments [26,37]. Similarly, but with a greater emphasis on providing research and support, others argue that the national level should identify priority areas while the local level carries out adaptation in practice [24,26,38,39]. Some authors have pointed to resource-provision as a primary purpose of national governments in adaptation [23,24]. Some additionally highlight the coordinating role of national governments in adaptation among sub-national levels of government [40,41]. A point of convergence in these debates is the need for national governments to take some form of action on adaptation, as its absence can be a constraint in itself to local-level adaptation and can contribute to inequitable levels of adaptation at sub-national levels [38,42].

In practice, the role a national government may take in adaptation will be determined by many factors but particularly by their constitutionally defined governing structure [42,43]. Determining national responsibility for health adaptation and allocating responsibility for adaptation planning and financing can be particularly challenging where public health is primarily the responsibility of sub-national governments, as in most decentralized and federal countries [28,44,45]. Governing structure thus provides a useful point of analysis in researching public health adaptation to climate change. We draw on Lijphart’s classifications of federal/unitary and decentralization/centralization structure based on whether there is a formal (federal) constitution, and how powers are dispersed across scales [46]. Though there is a spectrum within each of these categories [46], they nonetheless provide us with a useful starting point. In countries with federal, decentralized structures (where public health is typically primarily the responsibility of sub-national governments), national governments are expected to adopt a more supportive approach to adaptation and implement groundwork initiatives for capacity building and research, while national governments in unitary, centralized systems will implement more adaptation actions and take a stronger steering role [47]. Misalignment of adaptation policy initiatives with the traditional governing structure is often deemed problematic as it reduces coherency and consistency to deal with public health adaptation, and limits the likeliness that policy will be implemented successfully. In this study we examine national governments’ approaches to public health adaptation, considering differences in governance structure. 

The public health sector’s *approach* to climate change adaptation has also been debated in the literature. Hess et al. identify two contrasting views on how climate change will impact health and how public health should respond to climate change [48]. The first view suggests that climate change will amplify existing public health threats, but increased investments to existing extensive public health infrastructure (e.g., program expansion) alongside adequate funding and support will be sufficient to manage the projected health impacts of climate change [16,48]. The argument here is that the public health field is relatively well equipped to adapt to climate change due to its similarity to conventional health care and public health practices [16]. Meanwhile, the second view argues climate change may also impact health through entirely distinct pathways, for instance by destabilizing systems supporting public health or threatening infrastructure, thus necessitating new and innovative responses [48]. While the two are not mutually exclusive, they can be contradictory, as too much confidence in conventional health care systems could increase ignorance of climate risks and reduce willingness to explore the alternative discourse. The prevailing argument taken in a particular context will impact funding priorities, prioritization of adaptation, infrastructure and preparedness decisions, and adherence to long-term approaches to public health [48,49].

Three key governance dimensions of public health adaptation have received greater consensus in academic literature: cross-sectoral collaboration, vertical coordination, and national adaptation planning [23,25,50,51,52,53]. These three dimensions stand out in the literature as highly necessary, pertinent to public health adaptation challenges, and applicable across country contexts. First, the health risks posed by climate change are cross-sectoral in nature, impacting health through multiple pathways, and involving a diversity of actors with varying roles and responsibilities, and different types of communities and populations with diverse vulnerabilities [50]. One of the primary recommendations from the 2015 Lancet Commission on Climate Change and Health is for governments to facilitate collaboration between ministries of health and other departments, such as environment [12]. A multi-sectoral approach is required to address multiple drivers of adverse health outcomes of climate change and identify the most effective and efficient interventions. Second, as discussed above, national governments play a key role in coordinating or facilitating adaptation across scales and are uniquely positioned to carry out a variety of influential roles [38,40,54]. Without coordination, adaptation may take an isolated and piecemeal approach thereby increasing the chances of maladaptation (increasing vulnerability to climate change in the long term or of other sectors, social groups or systems) [55,56]. Climate change requires coordination of demands and needs across scales, to create synergies and avoid trade-offs between scales and at minimum to clarify jurisdictional roles and responsibilities in adaptation [37,38,39]. Other studies find most national governments have not taken a large leadership or steering role to date, and national adaptation policies are often disconnected from sub-national levels [39,40,41,42]. Lastly, national adaptation planning can serve to overcome challenges of horizontal and vertical coordination, provide a coherent and consistent public health adaptation policy, and enable and include private sector adaptation to invest resources (e.g., time, money) if or when needed. National adaptation plans may also be used to clearly outline the roles and responsibilities of sectors, agencies or scales, create consistency and synergy between national strategies, or meet regional adaptation planning requirements [42,44]. In this study we identify practical options for national governments to incorporate these three key governing dimensions into national-level public health adaptation planning in different contexts based on country experiences.

A small number of papers have begun tracking national-level adaption to the health impacts of climate change [24,28,30,57], but in general little is known about how adaptation is occurring in practice. Lesnikowski et al. examined national-level adaptation to the health impacts of climate change in Annex I countries to the UNFCCC (i.e., high income nations) by reviewing adaptation initiatives reported in countries’ Sixth National Communication to the UNFCCC (NC6) [24]. They present aggregated results and find that adaptation is piecemeal and mostly composed of groundwork initiatives, and that major health vulnerabilities are not being addressed by countries [24]. In a follow-up article, Lesnikowski et al. find national wealth, population size, perception of corruption, national environmental governance, and engagement in international environmental governance to have a statistically significant relationship with national-level health adaptation planning, suggesting that population size and national wealth are not sufficient to drive adaptation [58]. Panic and Ford conducted a systematic web search method to examine national-level adaptation planning in relation to infectious disease risks in 14 OECD countries, examining adaptation plans more broadly than specific initiatives [30]. They also find adaptation planning to be an ad hoc and fragmented process, and note three primary gaps based on best practices identified in academic literature: consideration of vulnerable populations, emphasis on local risks, and logistics (i.e., how adaptation will be carried out in practice) [30]. Our approach, presented here, combines Lesnikowski et al.’s focus on individual health adaptation initiatives with Panic and Ford and Bauer et al.’s examination of national adaptation plans [23,24,30]. Whilst some progress has been made on understanding how public health adaptation takes place and which role structural governing dimensions play, considerable questions remain which we address in the remainder of this paper.

## 2. Materials and Methods

We systematically review public health adaptation to climate change by national governments in ten OECD countries using publicly available information in government documents and websites. Conceptually and methodologically, we seek to track and assess adaptation in a comparable, consistent, comprehensive and coherent manner—the ‘4Cs’ of adaptation tracking [18,59]. This provides the basis not only for characterizing the current state of health adaptation reporting, but also for monitoring, evaluating, and communicating adaptation over time [18,60].

### 2.1. Sample

We included national governments as our comparable unit of analysis, selected based on OECD membership and on having official languages in either English or French (opportunistic sampling based on the languages spoken by the research team): Australia, Belgium, Canada, France, Ireland, Luxembourg, New Zealand, Switzerland, the United Kingdom (UK) and the United States (US). In the UK, responsibility for adapting to climate change has been devolved to national authorities in Scotland, Wales and Northern Ireland [61]. We included any reported adaptation initiatives that were implemented across the entire UK or in England, since Her Majesty’s Government is responsible for adaptation initiatives at both levels, consistent with other studies [23]. Limiting our sampling to these ten countries sacrifices the potential comprehensiveness of sampling all countries and the ability to make broader inferences; however, this smaller sample allows us to examine each country’s context in more depth and conduct a qualitative assessment of adaptation, while still providing sufficient data to make inferences for similar OECD countries [18].

### 2.2. Data Collection

To consistently and systematically locate health adaptation initiatives in national policy documents, national adaptation plans, and government websites, in March 2015 we conducted systematic web searches and assessment of self-reporting, combining two approaches used in other adaptation tracking studies [24,28,57,62,63]. We developed search strings tailored for Google web searches in English and French (see Table S1 of Supplementary Materials). We reviewed the first 30 search results, then examined further results until we had reached 30 consecutive irrelevant results, consistent with other adaptation tracking studies [28,30]. We then conducted reference tracking of the reviewed documents, reviewed each country’s NC6, and hand-searched each country’s equivalent of Ministry of Health, Ministry of Environment, and climate change commission(s) (where applicable). These methods combine Lesnikowski et al.’s approach of reviewing countries’ NC6 for data on adaptation, and Panic and Ford’s systematic web search method and review of adaptation planning documents [24,30]. These methods and use of multiple data sources allowed for triangulation and the development of a comprehensive dataset, while maintaining a systematic approach to data collection [18,24,64].

We conducted content analysis to both qualitatively and quantitatively review the health adaptation initiatives reported in the government documents and webpages retrieved in the systematic web search. Content analysis is a commonly used research method in the social sciences, one form of which allows the researcher to review secondary sources and count the frequency of a phenomena, in this case public health adaptation initiatives [65,66]. Adaptation initiatives from webpages and documents were only included if explicitly described as adaptation to climate change and referred to as adaptation to protect human health. As such, initiatives such as infectious disease surveillance, which may already be designed for surveillance of changing and emerging infectious diseases, were excluded if not described as being an adaptation, despite potential contribution to increasing resilience or reducing vulnerability to climate change. Similarly, although adaptation initiatives in other sectors may indirectly also protect health, these were excluded if not explicitly intended to protect health for consistency in data collection and feasibility. These methods are consistent with other studies in this area [24,28,30,41,57,64,67,68] and reflect the challenge of evaluating and defining programs as adaptations unless self-identified as such [60]. See Table S2 of Supplementary Materials for document inclusion criteria.

### 2.3. Indicators

For each initiative, we collected indicators aiming to coherently assess the quality of adaptation based on the Intergovernmental Panel on Climate Change (IPCC)’s Fifth Assessment Report [69] and academic literature [24,70], and going beyond simply documenting the number of initiatives [18]. Discrete health adaptation initiatives were documented in a detailed spreadsheet and coded according to the following measures adopted from other studies [24,70,71]: year, implementing body, adaptation type, health risk addressed, and consideration of vulnerable groups. Adaptation type is broken down through two different classifications. The first refers to either *groundwork initiatives* which build adaptive capacity, prepare the conditions for adaptation, or enable adaptation actions; or *adaptation actions* which indicate action has actually been taken to reduce population’s health vulnerability or increase resilience [24]. The second adaptation typology classifies adaptation initiatives by capacity building, management planning and policy, practice and behaviour, information, and warning or observing systems (Table 1) [70].

### 2.4. Tracking Reporting of Adaptation

Identifying data for comparison of adaptation across countries remains a significant challenge for tracking adaptation across nations [18,19]. Data on adaptation are difficult to find, and thus we must rely on the *reporting* of adaptation as the best option currently available for systematic analysis [19,64]. Reporting of adaptation in itself is one of several important proxies for prioritisation of adaptation and adaptive capacity [28,57,64]. In this research we are searching for adaptation activities that are framed by the responsible authority as adaptation to climate change. Limited reporting may hinder sharing of experiences and best practices, learning, transparency, and effective monitoring and evaluation [19,28,72,73]. Accordingly, in this paper we are comparing reporting of actions, rather than comparing actions per se, consistent with work assessing policy progress for other health issues [74,75].

## 3. Results

In total, 175 discrete health adaptation initiatives were identified and reviewed in 53 government documents or webpages (see Table S3 of Supplementary Materials). Countries with smaller populations (e.g., Ireland, Luxembourg, New Zealand) reported fewer health adaptation initiatives than countries with larger populations with the exception of Australia which has a relatively large population and high Gross Domestic Product (GDP) but reported few health adaptation initiatives (Table 2). These findings are mostly consistent with Lesnikowski et al.’s study which finds that population size and GDP are significant drivers of health adaptation [58]. Almost all of the documents reviewed (containing 94% of the total initiatives identified) were released in 2010 or later, following the same trend as adaptation in other sectors [21]. The earliest government document reviewed was published in 1995. It is possible more health adaptation initiatives were developed earlier but were not published online, were dismantled, replaced, or removed from the internet. For example, the UK’s 2012 health vulnerability assessment references an earlier health vulnerability assessment that is no longer available online, and thus not included in this study [76].

### 3.1. National Adaptation Planning Typically Does Not Target Specific Health Risks

**Figure 1:**
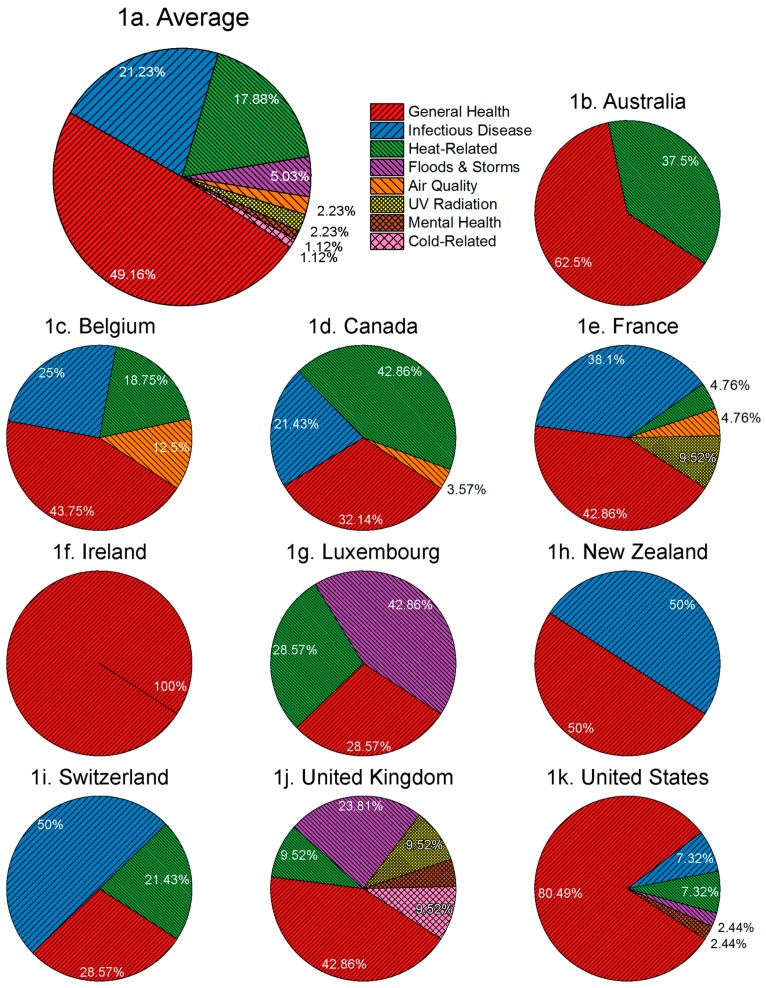
Percentage of health risks addressed by identified health adaptation initiatives.

National governments most frequently report planning broad health adaptation initiatives, and otherwise emphasize infectious disease and heat-related risks. Nearly half of the health adaptation initiatives planned or implemented by national governments do not target specific health risks, likely due to the nature of national-level initiatives and policies intended to guide more targeted actions at the sub-national level (Figure 1) [28,79]. The most frequently addressed health risks are infectious diseases (*n* = 38) and heat-related risks (*n* = 32). All of the sampled countries will or are experiencing changing incidence, prevalence or patterns of food-, water-, or vector-borne infectious diseases [71]. For example, in northern Canada climate change is projected to increase the incidence and prevalence of food- and water-borne infectious diseases among indigenous communities, and in New Zealand climate change could allow new mosquito vectors to establish resulting in changing patterns of existing and emerging vector-borne infectious diseases [80,81]. Similarly, the emphasis on adaptation to protect populations from heat-related risks may be in response to the high level of mortality from recent heat waves in the sampled countries. Whereas we find national governments are planning or implementing fewer initiatives specifically targeting floods and storms (*n* = 9), air quality (*n* = 4), ultraviolet (UV) radiation (*n* = 4), mental health (*n* = 2) or cold-related risks (*n* = 2) (Figure 1), a study of sub-national health adaptation found that local governments are targeting flood and storm risks and heat-related risk, but not addressing infectious disease risks [57]. This variation suggests national-level governments are planning or implementing more population-level health adaptation initiatives, while local governments’ health adaptation initiatives are more targeted to the local/regional level. Initiatives addressing the mental health risks associated with climate change have only been planned or implemented since 2013 within our sample. Despite recently receiving greater recognition in the academic literature [5,82], mental health and associated adaptation policy action received minimal attention in reporting on adaptation among the sampled countries.

### 3.2. National Governments Report Planning or Implementing Primarily Groundwork Adaptation Initiatives

**Figure 2:**
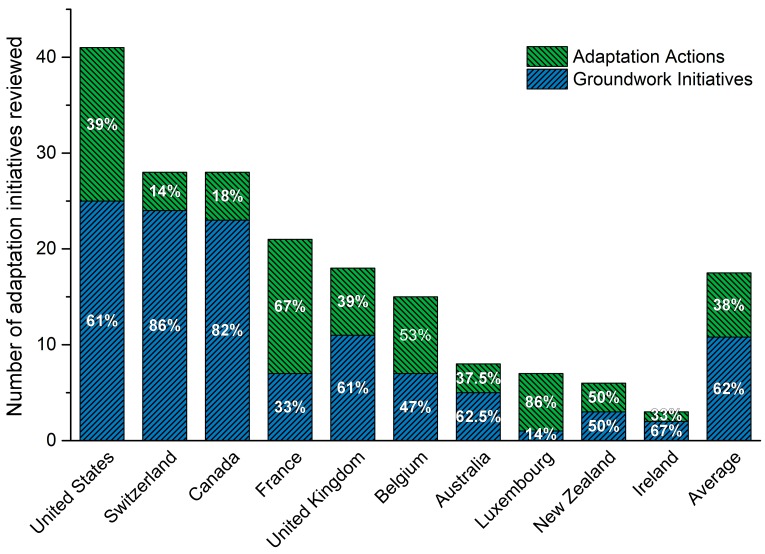
Percentage of groundwork initiatives and adaptation actions by country, and number of health adaptation initiatives identified by country.

**Figure 3:**
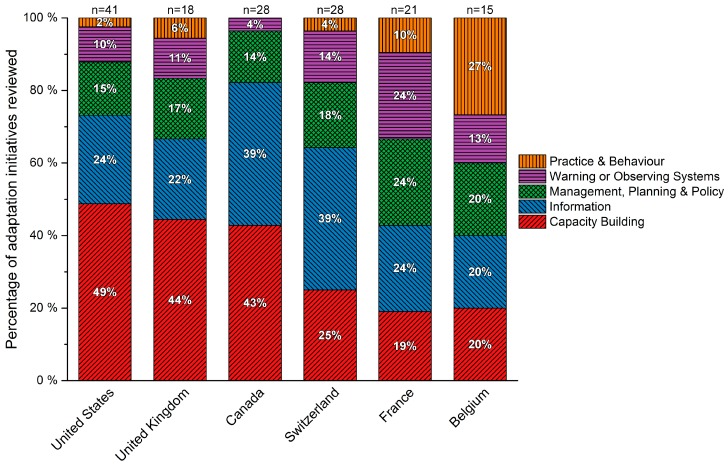
Percentage of adaptation type by sub-sampled country. Countries with less than 15 identified adaptation initiatives (Australia, Luxembourg, New Zealand and Ireland) are excluded from this figure. Descriptions of the adaptation types are available in Table S4 of Supplementary Materials.

National government adaptation initiatives were, averaging across countries, 62% groundwork and 38% adaptation actions (Figure 2). The exceptions are France, Belgium and Luxembourg, which report planning or implementing >50% adaptation actions each. Meanwhile, most countries reporting >10 initiatives are planning or implementing some combination of all five adaptation types (Figure 3). In Canada and the UK, capacity building initiatives dominate, and are predominantly guidebooks, frameworks or toolkits, while in the US these initiatives are a combination of training and professional education, guidebooks, frameworks or toolkits, and dissemination. France and Belgium report implementing or planning the highest number of practice and behaviour initiatives, including initiatives to adapt the techniques used in building health facilities (France) and eradicate the *Aedes japonicus* mosquito vector (Belgium). See Table 1 and Table S4 of Supplementary Materials for further examples of each adaptation type. Countries of contrasting administrative structure (federal and decentralized, or unitary and centralized) (Table 2) report similar adaptation approaches. The UK and the US, for example, report similar groundwork vs. adaptation initiatives (Figure 2) despite greater centralization in the UK. Likewise, Belgium and France show similarity in adaptation types being planned or implemented (Figure 1), despite contrasting administrative structures. Indeed, some of the sampled countries with federal, decentralized administrative structures are taking central roles in coordinating adaptation across jurisdictional levels, such as Belgium and Switzerland, through legislation and coordinated adaptation planning.

### 3.3. The Role of Inter-Sectoral Adaptation Planning Varies across Countries

Based on publicly available information, most adaptation initiatives were planned, initiated or implemented by national health or public health agencies, often in partnership with other bodies (including other health agencies). France, Belgium, the UK and New Zealand report the greatest percentage of health adaptation initiatives (~50%) being implemented (or planned) by, or in collaboration with, agencies or departments outside of the health sector (e.g., forestry sector, water resources sector, built environment sector, defense sector). In France, for example, the initiative to monitor vectors and host reservoirs is a collaboration between four health agencies and the French Research Institute for Exploitation of the Sea. Meanwhile in Canada, the US, and Switzerland, the vast majority of reported health adaptation initiatives are being planned, initiated and/or implemented solely within the health sector (82%, 78% and 69%, respectively). Though reporting a lower percentage of health adaptation initiatives conducted with other sectors, Switzerland is the only country reviewed to include animal health in the health section of its national adaptation plan, with significance for evaluating zoonotic disease risks [83]. Table S5 of Supplementary Materials lists the departments, agencies or other bodies involved in planning or implementing the health adaptation initiatives reviewed in each country.

### 3.4. National Adaptation Frameworks Provide Important Reference Points for Strategic Action

All sampled OECD countries have publicly available adaptation planning documents, with the exception of New Zealand. Of the nine national governments with a national adaptation planning document, only Canada and Ireland’s documents do not include explicit health sections [29,84]. Ireland’s adaptation framework states that sectoral adaptation plans would be completed by mid-2014 [29]; however a health sector plan has not yet been developed [85]. Canada’s National Adaptation Policy Framework does not include any sector-specific sections or specific adaptation initiatives, but rather serves to outline the federal government role in adaptation [84]. For some countries adaptation planning documents serve to identify adaptation objectives and guide future directions, such as in Australia [86], while for others adaptation plans are more detailed action plans, such as in Switzerland’s adaptation plan, which outlines responsible agencies and financing for each initiative [83]. Most of the sampled countries reporting a high number of public health adaptation initiatives have national legislation (or executive orders in the case of the US) for climate change which may have contributed to progress on adaptation planning (i.e., UK Climate Change Act (2008); Belgium Climate Cooperation Agreement (2002); Switzerland CO2 Act (2013); France Law n° 2009–967 (2009); US Executive Orders 13514 (2009) and 13653 (2013)). Meanwhile, New Zealand’s Resource Management Act Section 7 (i) was amended in 2004 to legally devolve statutory responsibility for consideration of climate change to local governments, which may explain in part the low number of reported national-level health adaptation initiatives. Table S6 of Supplementary Materials provides background on each country’s adaptation planning and health system.

## 4. Discussion

We examined the state of health adaptation in 10 OECD countries, based on systematic web searches and self-reporting of adaptation. Our findings demonstrate that some countries have begun to show evidence of comprehensive, strategic public health adaptation planning. Nevertheless, there are still significant steps to be made both in groundwork and health adaptation action [22,64,87]. National governments are central actors in public health adaptation and are well positioned to play a key role in national adaptation planning [26,40]. Developed countries’ national governments have high adaptive capacity, resources and human capital, but these do not necessarily translate into adaptation itself, and health adaptation in OECD countries remains varied. National governments are often constrained by existing institutional arrangements, such as conflicting mandates or fragmentation, or low political or public prioritization of climate change [43,88]. Moreover, there remains a potential gap between planned adaptation discussed in adaptation strategies and if and how the adaptation initiative is implemented in practice [89]. In this section we discuss the roles national governments have taken in public health adaptation to climate change based on our findings in relation to governing structure, identify options for approaches to public health adaptation that incorporate cross-sectoral collaboration, vertical coordination and national adaptation planning, and discuss this study’s limitations.

### 4.1. Governing Structure and National Governments’ Roles in Public Health Adaptation

We find national governments are taking a variety of roles in public health adaptation in practice, though the variation in approaches does not simply follow the federal, decentralized/unitary, centralized divide. Among the sampled federal countries for example, the Swiss and Belgian national governments have both primarily taken central coordinating roles in adaptation, employing legislative policy instruments to formalize vertical coordination with the cantons and regions, respectively [83,90,91,92]. The Swiss government has also prioritized capacity building for example through its health adaptation guidelines for Swiss municipalities [83,93], while the Belgian government has focused on planning or implementing national-level surveillance and programs (e.g., eradication of *Aedes japonicus* mosquito, infrastructure maintenance) [94]. Coordination is not prominently stressed, however, in the US’ or Canada’s health adaptation planning documents and webpages. The US has sought to put adaptation on the national policy agenda through recent executive orders requiring adaptation planning among national agencies [95], but has also emphasized resource provision and capacity building, most notably through the Building Resilience Against Climate Effects (BRACE) Framework and Climate-Ready States and Cities Initiative [96,97]. The Canadian national government’s approach has been to identify priority areas for action through extensive vulnerability assessments and research [98,99], and provide resources such as a series of extreme heat events guidelines [100,101,102], then allow sub-national governments and health officials to adopt adaptation initiatives as they see fit. Meanwhile, among the sampled unitary systems, the UK’s approach has prioritized cross-sectoral collaboration on adaptation, while also emphasizing capacity building and identifying priority areas for action [103]. The French national government has identified priority areas for health adaptation action, and conducted centrally-driven surveillance and adaptation of hospital infrastructure [104,105]. These varied approaches to national-level health adaptation, along with the types of adaptation initiatives being planned or implemented (see Section 3.2) suggest that though federal governments are taking a larger coordinating role, the federal/unitary divide does not fully explain the variation in national-level approaches to public health adaptation.

### 4.2. Options for Cross-Sectoral Collaboration, Vertical Coordination and National Health Adaptation Planning

A ‘one size fits all’ set of criteria is not appropriate for adaptation [106]. Based on our findings and insights from academic literature we outline options and approaches for cross-sectoral collaboration, vertical coordination and national health adaptation planning for national governments in countries with different state structures, jurisdictional contexts and administrative traditions. While many other best practices for health adaptation have been identified in the academic literature, these three dimensions were selected due to the consensus for their need across academic literature, for their pertinence to public health adaptation challenges, and most importantly for their flexibility and applicability across country contexts.

Our results show national governments may identify the form (policy integration) best suited to their country’s political context and governance system so as to increase legitimacy and policy effectiveness of public health adaptation initiatives [107,108]. Cross-sectoral collaboration may take many different forms, including top-down government driven forms of collaboration or a horizontal governance approach incorporating many actors through network steering [25]. Our review of national adaptation plans and other academic literature suggest other sectors (e.g., environment, natural resources) or climate change commissions (or equivalent) most often tend to take the lead coordinating role in national adaptation [22]. Health agencies can thus collaborate with coordinating sectors on national adaptation planning or actively collaborate with other sectors on a policy or program level. We find that the UK and France have notably included multiple sectors in health adaptation initiatives. The UK has taken a partnerships approach to adaptation and cross-sectoral collaboration, illustrated, for example, by the UK Natural Hazards Partnership, which prepares a daily hazard assessment (early warning system) and is comprised of thirteen diverse bodies including the Health Protection Agency, the Environment Agency and the UK Space Agency [103,109]. In France, cross-sectoral collaboration and collaborative networks have been instrumental in their monitoring and surveillance adaptation initiatives [104]. Bowen and Ebi have also identified creating collaborative networks, demonstrating leadership, integrating across organizations, scales and sectors, and coordinating government policies as additional approaches to cross-sectoral collaboration [25]. Challenges to cross-sectoral collaboration identified in the literature include: differing agency mandates and responsibilities; clarifying institutional arrangements to bridge gaps between sectors; and identifying sufficient funding sources, as funding typically comes from within sectors [26].

As we have discussed, national governments are taking different roles in public health adaptation. Our results and the literature likewise show that vertical coordination may take many forms, including institutionalized coordination bodies (e.g., working groups), network and partnerships, informal communication channels, monitoring and reporting schemes, and temporary coordination and consultation for elaborating national adaptation plans [23,87]. Belgium and Switzerland, for example, have both developed legislative mechanisms for vertical coordination [7,45]. In 2011, the Swiss federal government implemented the *loi sur le CO_2_* (CO_2_ Act) (updated in 2013) which includes an article requiring coordination of adaptation measures [92]. The Swiss adaptation action plan clearly lays out that the national government will work with sub-national governments and which sectors will work together on cross-sectoral issues [83]. In Belgium the *Commission Nationale de Climat* (CNC) (National Climate Commission), comprised of federal and regional representatives, was created as part of Article 3 of the 2003 Climate Cooperation Agreement to implement and monitor the Agreement, to harmonize the climate policies developed by the federal and regional governments, and to create synergies between them [90,91]. The US Centers for Disease Control and Prevention (CDC) directly assists selected state and local governments in preparing for the health effects of climate change through the Climate-Ready States and Cities Initiative [97,110]. Meanwhile, the US Department of Health and Human Services supports or collaborates with sub-national governments on several adaptation initiatives, employing a less formal approach [13]. Public health literature also suggests collaborative relationships and consensus building through inter-governmental agreements is one approach that could reduce risk of jurisdictional infringement [46].

Lastly, national adaptation plans may take different forms to suit different country contexts and institutional arrangements, and may be undertaken within and by a particular agency or sector, through the formation of a cross-cutting working group or committee, or by external bodies [53]. National adaptation plans may be comprehensive (e.g., France’s *Plan national d’adaptation au changement climatique* (national climate change adaptation plan) [104], the UK’s National Adaptation Programme [103]) or sector-specific (e.g., US Department of Health and Human Services Climate Adaptation Plan [96]). This distinction is important in ensuring health issues are sufficiently included in comprehensive plans which are typically coordinated by environment or natural resources ministries [87], and that horizontal coordination is incorporated into sectoral plans. As with vertical coordination, preparing a jurisdictionally appropriate national adaptation plan is particularly challenging in federal, decentralized countries, where sub-national governments may be responsible for health. In federal contexts an adaptation plan may thus be broad rather than very specific. The US’s Department of Health and Human Services, for example, does not steer sub-national adaptation in its adaptation plan, but outlines how the Department will adapt its own activities and work with other agencies and sub-national governments [96]. Belgium has taken another approach to national adaptation planning in a federal, decentralized context, where the National Climate Commission (comprised of representatives from all government levels) will use the federal and regional government adaptation plans as a basis for a future national adaptation plan, thereby using national adaptation planning as a tool to engage sub-national governments [52,91,111].

Countries are developing their own policy style, learning from key lessons ascertained in other countries as well as mimicking successful approaches. Whilst constitutionally defined governing structures are important features in the ways public health adaptation are governed, they do not determine the ways in which public health adaptation takes place, therefore showing a great variety of policy initiatives, both substantive as well as procedural. This is not necessarily a bad thing—countries are clearly experimenting with different ways how to best govern public health adaptation across sectors and levels, as the diversity and rapid increase of policy initiatives suggests. Tracking this progress then becomes of utmost importance to assess and evaluate whether progress is going in the right direction. 

### 4.3. Limitations

Using publicly available government documents, this study has examined the reporting of health adaptation as a basis for examining the current state of adaptation in a health context, using an approach consistent with other studies tracking adaptation [24,28,30,41,57,63,64,67,68]. This method poses some challenges to comprehensiveness of the dataset as policies or programs that reduce vulnerability or increase resilience to climate change may be underreported or not labeled as adaptation, and not captured by the search methods. However, initiatives that are not intended as adaptation will not include projected or perceived climate change impacts as the starting point for decision-making and these initiatives risk being maladaptive [112]. 

This method also relies exclusively on primary sources, thus we cannot draw conclusions on why national governments decide to frame or design their policies in the reported manner, or what their intentions are in regards to climate change adaptation planning other than explicit objectives. Moreover, some of the national adaptation plans reviewed do not include explicit plans for how adaptation initiatives will be implemented, demonstrating that we can only review the reporting of health adaptation, not the implementation, output or outcome. Though reporting of adaptation is an imperfect proxy and subject to bias, there are currently few alternatives that provide the level of detail needed to track health adaptation activities across multiple countries [18].

Though the importance of considering multiple scales has been highlighted in climate change adaptation literature [36,113], in this study we have examined exclusively national-level public health adaptation. Sub-national governments are important players in adaptation, particularly where some may have the jurisdictional mandate for public health and adaptation; however, their large number makes them outside the scope of this study. We do not make claims to have tracked public health adaptation in the sampled countries beyond the national government, but have examined the ways in which governments create an enabling environment. Focusing exclusively on national governments, however, allows us to compare and contrast national-level approaches to public health adaptation, consider variations with governing structure, and identify possible adaptation options for national governments.

## 5. Conclusions

Climate change and the associated public health risks are a formidable challenge for health officials, and national governments have been identified as key players in public health adaptation, yet little research has empirically tracked national-level public health adaptation to climate change across multiple countries [24,30]. In this study we have systematically assessed how national governments report adapting to the health risks posed by climate change, reviewed national governments’ roles in public health adaptation and examined practical options for incorporating three key dimensions for national-level governance of public health adaptation—cross-sectoral collaboration, vertical coordination, and national health adaptation planning—across different country contexts.

Following the adoption of the Paris Agreement and greater momentum in the health community for action on climate change [11,12,17], national governments are beginning or continuing to evaluate potential public health adaptation options. These findings demonstrate that while some countries have yet to report prioritizing, planning or implementing public health adaptation, others can serve as models and provide a learning opportunity for governments to incorporate the key dimensions for national-level governance of public health adaptation. Tracking public health adaptation to climate change is crucial to improve understanding of how adaptation is occurring in practice and across states, and to ensure policy orientated learning.

## Figures and Tables

**Table 1:** Adaptation Typology.

Adaptation Category	Description	Examples of Initiatives in Category
Capacity Building	Developing human resources, institutions, and communities, equipping them with the capability to adapt to climate change.	Educate health professionals about the health impacts of climate change (FR, BE) Heat risk adaptation guidelines for public health and emergency management officials (CA) Raise awareness of climate change impacts and social vulnerability (IR)
Management, Planning and Policy	Incorporating understanding of climate science, impacts, and vulnerability and risk into government and institutional planning, management, policies and regulations.	Creation or strengthening of centers and networks of expertise at national and international levels (SW) Establishment of an internal multidisciplinary work group to investigate the occupational safety and health implications of climate change (US) Heat wave plan (UK)
Practice and Behaviour	Revisions or expansion of practices and on the ground behaviour that are directly related to building resilience.	Eradication of *Aedes japonicus* mosquito (BE) Analyze and adapt the techniques used in building health and social facilities (FR) Stockpile critical medical supplies and pharmaceuticals (US)
Information	Systems for communicating climate information to help build resilience towards climate impacts (other than communication for early warning systems).	Identify the capacity of the public health system and hospital system to plan and respond to vulnerabilities (AU) Assess vulnerabilities and health impacts of climate change among Northern/Inuit populations (CA) Research the potential effects of weather patterns and climate on outbreaks of environmentally-sensitive infectious diseases (US)
Warning or Observing Systems	Implementation of new or enhanced tools and technologies for communicating weather and climate risks, and for monitoring changes in the climate system.	Surveillance for heat response plan (LU) Food- and water-borne infectious disease surveillance (NZ) Maintain and expand real time UV monitoring (UK)

Note: The following abbreviations refer to the country’s national government planning or implementing the example health adaptation initiative: Australia (AU), Belgium (BE), Canada (CA), France (FR), Ireland (IR), Luxemburg (LU), New Zealand (NZ), Switzerland (SW), United Kingdom (UK), United States (US). Table adapted from “A typology of adaptation actions: A global look at climate adaptation actions financed through the Global Environment Facility” by Biagini et al., 2014, Global Environmental Change.

**Table 2:** Country Information.

Country Name	Population Size (2015) [77]	GDP (Billion USD) (2014) [78]	GDP/Capita (PPP Adjusted) (2015) [78]	Governing Structure [46]	Number of Health Adaptation Initiatives Reviewed
Australia	23,490,736	1454.7	45,514.2	Federal and decentralized	8
Belgium	11,225,207	531.2	43,991.6	Federal and decentralized	15
Canada	35,540,419	1783.8	44,310.1	Federal and decentralized	28
France	66,206,930	2829.2	39,678.0	Unitary and centralized	21
Ireland	4,612,719	250.8	54,654.4	Unitary and centralized	3
Luxembourg	556,074	64.9	101,926.4	Unitary and centralized	7
New Zealand	4,509,700	200.1	36,982.3	Unitary and centralized	6
Switzerland	8,190,229	701.0	60,535.2	Federal and decentralized	28
United Kingdom	64,510,376	2990.2	41,324.6	Unitary and centralized	18
United States	318,857,056	17,348.1	55,836.8	Federal and decentralized	41

Note: GDP refers to Gross Domestic Product, and PPP refers to Purchasing Power Parity.

## Supplementary Materials

The following are available online at www.mdpi.com/1660-4601/13/9/889/s1, Table S1: Example web search strings used for locating national health adaptation initiatives, Table S2: Inclusion and exclusion criteria for health adaptation documents, Table S3: Adaptation planning documents or government webpages included, Table S4: Public health adaptation initiatives reviewed by adaptation type or sub-category of adaptation type, Table S5: Departments, agencies or other bodies involved in planning or implementing health adaptation initiatives reviewed, Table S6: Background information on included countries’ adaptation planning and health system.
