# Supplementary material for: Public Health Adaptation to Climate Change in OECD Countries
Source: Int J Environ Res Public Health. 2016 Sep 7;13(9):889. doi: 10.3390/ijerph13090889 (PMC5036722; doi:10.3390/ijerph13090889)
Supplement: Supplementary file 1 [file ijerph-13-00889-s001.pdf]

Supplementary Materials: Public Health Adaptation to Climate Change in OECD Countries

Stephanie E. Austin, Robbert Biesbroek, Lea Berrang-Ford, James D. Ford, Stephen Parker and Manon D. Fleury

Appendix A: Search Strings

We developed search strings tailored for Google web searches (Table S1), adapting Panic & Ford and Austin et al. [1,2]. Synonyms for each country and the country's government website domain were used for each country-specific search string. Web searches were conducted using country-specific Google searches (e.g., google.ca or google.fr).

Table S1. Example web search strings used for locating national health adaptation initiatives.

Language	Search String
English	("climate change" OR "global warming" OR "climatic change") health (adaptation OR adaption OR ~adapting OR ~coping OR response OR ~responding) (Canada OR Canadian OR "gc.ca")
French	("changements climatiques" OR "changement climatique" OR "réchauffement de la planète" OR "réchauffement planétaire" OR "réchauffement climatique") santé (adaptation OR ~adapte OR ~répond OR réponse) (France OR français OR "gouv.fr")

Appendix B: Document Inclusion and Exclusion Criteria

For documents to be included in our analysis, they had to be a governmental document or webpage in English or French; to have climate change as the overarching focus; to address human health adaptation initiatives; to include federal level initiatives; and to be a technical document, adaptation plan, national report, adaptation assessment, vulnerability assessment or government webpage (detailed in Table S2).

Table S2. Inclusion and exclusion criteria for health adaptation documents.

Inclusion Criteria	Exclusion Criteria
English or French language	Non-English or French language
Must contain at least one public health adaptation initiative, or constitute an initial in itself (e.g., a vulnerability assessment)	Does not contain any nor constitute a public health adaptation initiative
Human adaptation to climate change	Non-human adaptation to climate change
Jurisdictionally appropriate documents and initiatives (e.g., national-level documents)	Jurisdictionally inappropriate documents and initiatives (e.g., sub-national-level documents)
Government documents, government websites, or documents by government-established research organizations/networks or consultants hired by the government	Documents by non-governmental organizations, unaffiliated institutions, private companies or professional associations
Technical documents, adaptation plans, national reports, adaptation assessments, vulnerability assessments, government webpages	Editorials, meetings, conferences, presentations, abstracts, financial evaluations of climate change adaptation
Addresses risks posed to human health by climate change	Addresses climate change risks unrelated to health (e.g., forest management)
Documents or webpages published in March 2015 or earlier	Documents or webpages published after March 2015

Appendix C: Documents or Webpages Included

Following systematic web searches and based on the inclusion/exclusion criteria detailed in Appendix B, 55 documents containing one or more public health adaptation initiatives, or constituting an adaptation initiative in itself (e.g., a vulnerability assessment) were included for review (Table S3).

Table S3. Adaptation planning documents or government webpages included.

Country Name	Author	Year	Title (URL If Applicable)
Australia	Department of Climate Change and Energy Efficiency	2007	National Climate Change Adaptation Framework
Australia	National Climate Change Adaptation Research Facility	2012	National Climate Change Adaptation Research Plan: Human Health—Update 2012
Belgium	National Climate Commission	2010	Belgian National Climate Change Adaptation Strategy
Belgium	Service Public Fédéral Santé Publique, Sécurité de la Chaîne Alimentaire et Environnement	2013	Projet de Plan Fédéral: 'Adaptation aux changements climatiques'
Belgium	Service Public Fédéral Belge	2013	Sixième communication nationale sur les changements climatiques
Belgium	Service Public Fédéral Belge	2014	Votre avis sur le projet de Plan Fédéral Adaptation aux changements climatiques (http://www.belgium.be/fr/actualites/2014/news_votre_avis_sur_plan_federal_adaptation_aux_changements_climatiques.jsp)
Canada	Health Canada	n.d.	Acute care during extreme heat: recommendations and information for health care workers
Canada	Health Canada	n.d.	Community care during extreme heat: heat illness: prevention and preliminary care
Canada	Health Canada	n.d.	Health facilities preparation for extreme heat: recommendations for retirement and care facility managers
Canada	Natural Resources Canada	2004	Climate Change Impacts and Adaptation: A Canadian perspective
Canada	Natural Resources Canada	2008	From Impacts to Adaptation: Canada in a Changing Climate 2007
Canada	Health Canada	2008	Health Canada Climate Change Program (http://climatetelling.ca/health-canada-climate-change-program/)
Canada	Health Canada	2008	Human Health in a Changing Climate: A Canadian Assessment of Vulnerabilities and Adaptive Capacity
Canada	Office of the Auditor General of Canada	2010	Report of the Commissioner of the Environment and Sustainable Development to the House of Commons
Canada	Health Canada	2010	What's Being Done about Climate Change and Health in Canada—Adaptive Actions (http://www.hc-sc.gc.ca/ewh-semt/climat/adapt/actions-eng.php)
Canada	Health Canada	2011	Extreme Heat Events Guidelines: Technical Guide for Health Care Workers

Table S3. *Cont.*

Country Name	Author	Year	Title (URL If Applicable)
Canada	Health Canada	2011	Communicating the Health Risks of Extreme heat Events: Toolkit for Public Health and Emergency Management Officials
Canada	Health Canada	2011	Extreme Heat Events Guidelines: User Guide for Health Care Workers and Health Administrators
Canada	Health Canada	2011	Adapting to Extreme Heat Events: Guidelines for Assessing Health Vulnerability
Canada	Health Canada	2012	Heat Alert and response Systems to Protect Health: Best Practices Guidebook
Canada	Public Health Agency of Canada	2013	Preventative Public Health Systems and Adaptation to a Changing Climate Program (http://www.phac-aspc.gc.ca/hp-ps/eph-esp/pph-psp-eng.php)
Canada	Natural Resources Canada	2014	Canada in a Changing Climate: Sector Perspectives on Impacts and Adaptation
Canada	Health Canada and the Public Health Agency of Canada	2014	Federal Climate Change and Health Adaptation Initiatives (presentation)
France	L’Agence française de sécurité sanitaire des aliments (Afssa)	2005	Rapport sur l’évaluation du risque d’apparition et de développement de maladies animales compte tenu d’un éventuel réchauffement climatique
France	Ministère de la Santé	2007	Stratégie nationale d’adaptation au changement climatique
France	Ministère de la Santé, de la Jeunesse, des Sports et de la Vie associative, et Ministère de l’Écologie, de l’Énergie, du Développement durable et de l’Aménagement du Territoire	2008	Les effets qualitatifs du changement climatique sur la santé: Rapport de groupe interministériel
France	Institut de Veille Sanitaire	2010	Impacts sanitaires du changement climatique en France: Quels enjeux pour l’InVS?
France	Ministère de l’Écologie, du Développement durable, des Transports et du Logement	2011	Plan National d’Adaptation au Changement Climatique
Ireland	Environmental Protection Agency	2011	Integrating Climate Change Adaptation into Sectoral Policies in Ireland
Luxembourg	Ministre de l'Environnement et du Ministre de la Santé et de la Sécurité Sociale	2007	Réponse commune du Ministre de l’Environnement et du Ministre de la Santé et de la Sécurité Sociale à la question parlementaire N°1993 de Messieurs les députés Laurent Mosar et Marcel Oberweis
Luxembourg	Le Gouvernement du Grand-Duché de Luxembourg	2014	Sixth National Communication of Luxembourg under the UNFCCC
New Zealand	Ministry for the Environment	2001	Climate Change: Potential Effects on Human Health in New Zealand
New Zealand	Ministry for the Environment	2013	New Zealand’s Sixth National Communication
New Zealand	Ministry of Health	2015	Opening address of the public health response to climate change (https://www.beehive.govt.nz/speech/opening-address-public-health-response-climate-change-%E2%80%93-mitigation-adaptation-and-action-univ)
Switzerland	L'Office fédérale de l'environnement (OFEV)	2012	Adaptation aux changements climatiques dans les villes suisses
Switzerland	Confédération Suisse	2012	Adaptation aux changements climatiques en Suisse

Table S3. *Cont.*

Country Name	Author	Year	Title (URL If Applicable)
Switzerland	Organe consultatif sur les changements climatiques (OcCC)	2012	Les changements climatiques et la Suisse en 2050: Impacts attendus sur l'environnment, la société et l'économie
Switzerland	Office Fédéral de la Santé Publique	2014	Projet pilote adaptation aux changements climatiques «Effet des canicules sur la mortalité et mesures d'adaptation possibles» (http://www.bafu.admin.ch/ klimaanpassung/12575/13224/14031/index.html?lang=fr)
Switzerland	Confédération Suisse, Département fédéral de l'environnement, des transports, de l'énergie et de la communication (DETEC), and Office fédéral de l'environnement (OFEV)	2014	Adaptation aux changements climatiques en Suisse—Plan d'action 2014–2019
United Kingdom	Health Protection Agency	2012	Health Effects of Climate Change in the UK 2012
United Kingdom	Department of Environment, Food and Rural Affairs	2012	UK Climate Change Risk Assessment: Government Report
United Kingdom	Department of Environment, Food and Rural Affairs	2013	National Adaptation Programme
United Kingdom	Committee on Climate Change	2014	Managing climate risks to well-being and the economy
United Kingdom	Sustainable Development Unit, Public Health England, National Health Service (NHS) England	2014	Adaptation to Climate Change: Planning Guidance for Health and Social Care organisations
United Kingdom	Public Health England and Environment Agency	2014	Flooding: advice for the public
United States	Environmental Protection Agency	2006	Excessive Heat Events Guidebook
United States	Interagency Climate Change Adaptation Task Force	2011	Federal Actions for a Climate Resilient Nation
United States	Centers for Disease Control and Prevention (CDC)	2012	Climate and Health Program—CDC's Building Resilience Against Climate Effects (BRACE) Framework (http://www.cdc.gov/climateandhealth/BRACE.htm)
United States	Centers for Disease Control and Prevention (CDC)	2013	Climate and Health Program—About Our Program (http://www.cdc.gov/climateandhealth/about.htm)
United States	Department of Health and Human Services (HHS)	2014	HHS Climate Adaptation Plan
United States	Department of Health and Human Services (HHS)	2012	HHS Climate Change Adaptation Plan
United States	Department of Veteran Affairs (VA)	2014	U.S. Department of Veterans Affairs Climate Change Adaptation Plan

Appendix D: Health Adaptation Initiatives Classified by Adaptation Type

We classified each discrete public health adaptation initiative as one of five types of adaptation, adapted from Biagini et al. [3]: capacity building; management, planning & policy; practice & behaviour; information; and warning or observing systems. However, there is significant variation in the instruments or mechanisms used within each adaptation type. Table S4 shows all of the public health adaptation initiatives reviewed for the six countries with the highest number of initiatives reviewed broken down by sub-categories of the capacity building, information, and management, planning & policy adaptation types for each country. Practice & behaviour and warning or observing systems initiatives are also shown by country, but are not divided into sub-categories given the low number.

Table S4. Public health adaptation initiatives reviewed by adaptation type or sub-category of adaptation type.

	Capacity Building					
	United States	*Canada*	*Switzerland*	*France*	*United Kingdom*	*Belgium*
Training and professional education	1. Develop climate change adaptation training for Department of Health and Human Services (DHHS) employees<br>2. Code red heat alert training<br>3. Hold a department-wide briefing on climate change and health<br>4. Adaptation planning workshop<br>5. Mental health resiliency training programs (pilot)	1. Disseminate accredited course for medical doctors to improve their understanding of extreme heat and assist in the treatment of patients		1. Educate health professionals about the health impacts of climate change		1. Educate health professionals about the impacts of climate change
Guidebooks, frameworks and toolkits	1. Excessive Heat Events Guidebook<br>2. Community tree-planting decision-making tools<br>3. Building Resistance Against Climate Effects (BRACE) Framework<br>4. Adaptation guidebook for state and local health departments<br>5. Resource packet related to resilience of health facilities in a context of	1. Heat risk adaptation guideline for public health and emergency management officials<br>2. Heat risk guidelines for health care workers and health administrators<br>3. Heat risk technical guide for health care workers<br>4. Adapting to Extreme Heat Events: Guidelines for Assessing Health Vulnerability	1. Health adaptation guidelines for Swiss municipalities<br>2. Develop a guide on strategy development for heat wave management in the sectors of health and social affairs<br>3. Preparation of information and recommendations concerning the prevention of vector-		1. National Flood Emergency Framework for England<br>2. National Health Service (NHS) Emergency Planning Guidance (2005)<br>3. NHS Emergency Planning Guidance on planning the psychosocial and mental health care	

	climate change-exacerbated stressors<br>6. Public Health Preparedness Manual	5. Heat alert and response systems best practices guidebook<br>6. Climate change infectious disease toolkit<br>7. Acute care during extreme heat: recommendations and information for health care workers<br>8. Community care during extreme heat: heat illness: prevention and preliminary care<br>9. Health facilities preparation for extreme heat: recommendations for retirement and care facility managers	borne infectious diseases		of people affected by major accidents and disasters (2009)<br>4. Planning guidance for health and social care organisations: Adaptation to climate change	
Public outreach and education	1. Collaborate with Environment Protection Agency (EPA) and other federal partners in the region to promote climate change education and awareness	1. Provide assistance to communities to conduct vulnerability assessments and advise on heat-health messaging and communications		1. Educate the public about the health impacts of climate change<br>2. Educate consumers about food safety	1. Sharing information and promoting understanding of the risks to different vulnerable groups<br>2. Flooding: Advice for the public (leaflet)	1. Maintain and develop awareness/education (schools, public, restaurants) and communication (reinforce with vulnerable populations)<br>2. Public consultation to inform the Federal climate change adaptation plan
Dissemination of information to decision-makers and stakeholders	1. Develop a climate change communication and outreach strategy<br>2. Dissemination of webinar announcements and other related educational materials regarding the results of climate change	1. Establish a sharing network for public health and emergency management officials	1. Coordinate collaboration between the confederation and the cantons (in the context of exotic mosquitoes)<br>2. Participate in sharing information	1. Adapt communication tools for UV prevention to overseas territories	1. Promotion of climate/extreme weather preparedness and resilience within the local health and social care system	

	and health to the Medical Reserve<br>3. Sharing climate change educational materials with state Emergency System for Advance Registration of Volunteer Health Professionals (ESAR-VHP) programs and volunteers via e-mail, listserv and the Assistant Secretary for Preparedness & Response (ASPR) ESAR-VHP website on a rolling basis<br>4. Extreme weather, climate, and health: putting science into practice (meeting)<br>5. Disseminate webinars and educational materials regarding the effects of climate change on human health (Office of Minority Health (OMH))<br>6. Disseminate webinars and educational materials regarding the effects of climate change on human health (Office on Women's Health)<br>7. Promote and disseminate the latest climate change and health information to stakeholders on a rolling basis		platforms at the federal and cantonal level (in context of infectious diseases)<br>3. Involvement in specialized networks WHO and ECDC (European Centre for Disease Prevention and Control) (in the context of infectious diseases)		2. Supporting the Local Government Association's (LGA) Climate Local initiative	

Management, Planning & Policy

	United States	*Canada*	*Switzerland*	*France*	*United Kingdom*	*Belgium*
Alert and Response Systems		1. Pilot alert and response system to heat events<br>2. Pilot alert and response system to infectious diseases				1. Heat waves and ozone plan

Plans				1. Heat wave plan	1. Cold weather plans<br>2. Heatwave plans	
Incorporating climate science, impacts, risks and vulnerability into governmental and institutional planning				1. Strengthen the management of occupational risks induced by climate change<br>2. Take climate change into account in national prevention and care plans<br>3. Strengthen climate-health research<br>4. Networking of existing expertise and improving dialogue between the spheres of research, administration and practice		
Creating new bureaus, departments or groups	1. Development of a leadership forum with which to integrate climate change adaptation<br>2. Establishment of an internal multidisciplinary work group to investigate the occupational safety and health implications of climate change<br>3. Establishment of the disaster technical assistance center (DTAC)		1. Creation or strengthening of centers and networks of expertise at national and int'l levels	1. Create a "healthy climate" monitoring group within the Haut Conseil de la Santé Publique (HCSP)	1. Creation of Extreme Events and Health Protection Section (EEHPS)	
Policies and regulations			1. Legislation "Loi sur les épidémies": creation of a coordinating body "plate-forme zoonose"	1. Application of uniform criteria for heatwave alerts		1. Regulations for adaptation of buildings to ensure thermal comfort of buildings and their occupants (thermal

			2. Defining thresholds for triggering heat wave alerts in the fields of health care and social affairs			insulation, solar protection shutters, ventilation)<br>2. Changes in lifestyle during a heat wave (adapting opening hours of public and private institutions)
Programs	1. Waste/Wastewater Agency Response Network (WARN)<br>2. Participating in the efforts of the Environmental Justice Interagency Working Group to develop an interdepartmental approach to climate change adaptation in environmental justice communities<br>3. Disaster Research Response Project	1. Program for Climate Change and Health Adaptation in Northern First Nation and Inuit Communities<br>2. Enhance Heat Alert and Response System (HARS) programs in existing regions and expand into other new at risk communities and regions				
			**Information**			
	United States	***Canada***	***Switzerland***	***France***	***United Kingdom***	***Belgium***
Vulnerability assessment	1. Vulnerability assessments for climate-related environmental hazards<br>2. Special report on the impacts of climate change on human health in the United States<br>3. Identify the effects of climate change on children's health	1. Climate change impacts and adaptation: A Canadian perspective (NRCan)<br>2. Canadian assessment of vulnerabilities and adaptive capacity (HC)<br>3. From Impacts to Adaptation (vulnerability assessment)<br>4. Assess vulnerabilities and health impacts of climate change amount Northern/Inuit populations	1. Vulnerability assessment	1. Report on the evaluation of the risk of occurrence and development of animal diseases considering possible global warming<br>2. Health Vulnerability Assessment (2008)<br>3. Health Vulnerability Assessment (2010)	1. Climate change risk assessment<br>2. Health effects of climate change in the UK 2012	

		5. Climate change impacts and adaptation: A Canadian perspective				
Research	1. Establish baseline climate scenarios for department-wide use in vulnerability assessments and adaptation development<br>2. Identifying populations vulnerable to health impacts of climate change (Climate and Health Program)<br>3. Use NASA data and models to improve heat watch warning systems<br>4. Research to determine the potential effects of weather patterns and climate on outbreaks of environmentally-sensitive infectious diseases such as Lyme disease and West Nile virus infection<br>5. Series of exploratory projects to identify the impacts of climate change on the health and safety of workers, and the potential for health and safety training to prepare workers and their communities<br>6. Research evaluating the impact of climate change on the health of Arctic populations	1. Assessment of the burden of AGI and adaptation to climate change in the Canadian North<br>2. Evaluation of the impacts of climate change on food and water safety and public health outcomes<br>3. Projected burden of Lyme disease in Ontario<br>4. Public health and water-borne illness research tool<br>5. Establish links between the built environment, urban heat island and efforts to build healthy, liveable communities<br>6. Continue research on extreme heat and partner with Environment Canada on improved forecasting of heat events	1. Improved information on the health risks associated with misuse of food<br>2. Periodic evaluation of the need for action in the field of water hygiene<br>3. Examine the need to adapt further to heat waves based on recommendations<br>4. Pilot research on the effect of heat waves on mortality and possible adaptation measures	1. Evaluate the health risks linked with preserving quantities of water resources<br>2. Map bathing water at risk of quality degradation in extreme temperatures	1. Map flood risk to health and social care infrastructure part of the Strategic Health Asset Planning & Evaluation toolkit (SHAPE)<br>2. Managing climate risks to well-being and the economy	1. Strengthening food chain safety: research and controls<br>2. Monitoring of potential effects of climate change on health
Systems for communicating climate information	1. Development of health data and tools for the federal government's open data platform www.data.gov/climate		1. Encouraging the exchange of experiences between the cantons in			

	United States	Canada	Switzerland	France	United Kingdom	Belgium
			procedures during heat waves			
Information consolidation & Reporting			1. Documentation of the state of knowledge and targeted future research<br>2. Sporadic knowledge consolidation on research projects on vector-borne infectious diseases<br>3. Establish reporting on the epidemiological situation<br>4. Establish reporting on the situation of exotic mosquitoes<br>5. Explore the possibility to instate an obligation to notify cases of other vector-borne infectious diseases			1. Monitoring WHO work on climate change and health by environment and health experts
**Warning or Observing Systems**						
	United States	***Canada***	***Switzerland***	***France***	***United Kingdom***	***Belgium***
All Warning or Observing Systems Initiatives	1. National surveillance for infectious diseases<br>2. Healthcare Associated Infections and Influenza Surveillance System (HAISS)—biosurveillance tool<br>3. Integrated public health surveillance and early warning systems<br>4. Addition of climate change indicators to the Centers for Disease Control and Prevention (CDC) tracking network	1. Air quality and health index	1. Monitoring of microbiological contaminants<br>2. Monitoring by sample *Aedes albopictus* and *Aedes japonicus* along major traffic routes in order to detect spread to other parts of the country as soon as possible<br>3. Addition of seven vector-borne infectious diseases to	1. Health surveillance network (infectious disease)<br>2. Monitor vectors and host reservoirs<br>3. Monitor the development of toxin-producing microorganisms<br>4. Monitor pollens and mould<br>5. Extend UV index calculations to overseas territories	1. Maintain and expand real time UV monitoring<br>2. Daily hazard assessment (early warning system)	1. Monitoring exotic mosquitoes in Belgium<br>2. Monitoring pollen and fungal spores

			the list of mandatory reporting and surveillance by the Federal Office of Public Health (OFSP)<br>4. Improving the capacity of monitoring and surveillance			
**Practice & Behaviour**						
	United States	***Canada***	***Switzerland***	***France***	***United Kingdom***	***Belgium***
All Practice & Behaviour Initiatives	1. Stockpiles of critical medical supplies and pharmaceuticals		1. Creation of a central information and advice service in case of emergency event	1. Strengthen food safety<br>2. Analyze and adapt the techniques used in building health and social facilities	1. Plan and maintain resilient healthcare facilities	1. Adoption of measures to better care for vulnerable and precarious people<br>2. Mandatory reporting by physicians in case of new infectious diseases and vectors<br>3. Adaptation of infrastructure (transport, making water sources available in public spaces)<br>4. Eradication of *Aedes japonicus* mosquito

Appendix E: Departments, Agencies or Other Bodies

Table S5. Departments, agencies or other bodies involved in planning or implementing health adaptation initiatives reviewed.

Country	Departments, Agencies or Other Bodies
Australia	• Adaptation Research Grant Program (ARGP)<br>• Australian Health Ministers' Conference<br>• National Climate Change Adaptation Research Facility (NCCARF)<br>• National Health and Medical Research Council<br>• Sport and Recreation Ministers' Council
Belgium	• Conférence Interministérielle de l'Environnment<br>• Institut scientifique de Santé Publique<br>• Service Public Federal Santé Publique, Sécurité de la Chaine Alimentaire et Environnement<br>• Veterinary and Agrochemical Research Centre (CODA-CERVA)
Canada	• Environment Canada<br>• Health Canada<br>• Natural Resources Canada<br>• Public Health Agency of Canada
France	• Agence nationale de securite sanitaire de l'alimentation, de l'environnment et du travail (Anses)<br>• Direction Générale de l'Aménagement, du Logement et de la Nature<br>• Agences Régionales de Santé (ARS)<br>• Institut national de prévention et d'éducation pour la santé (Inpes)<br>• Institut de Veille Sanitaire (InVS)<br>• Direction Generale de la Sante (DGS)<br>• Le Reseau de Surveillance Aerobiologique (RNSA)<br>• Haut Conseil de la Santé Publique (HCSP)<br>• Institut francais de recherche pour l'exploitation de la mer (Ifremer)<br>• Ministère de la Santé<br>• Institut national de recherche en science et technologies pour l'environnement et l'agriculture (cemagref)<br>• L'Agence française de sécurité sanitaire des aliments (Afssa)<br>• Ministère de l'Enseignement Supérieur et de la Recherche<br>• Ministère de la Santé, de la Jeunesse, des Sports et de la Vie associative (*no longer exists*)<br>• Ministère de l'Écologie, de l'Énergie, du Développement durable et de l'Aménagement du Territoire

Ireland	• Department of Health and Children (DHC)<br>• Department of Social Protection (DSP)<br>• Health Research Board (HRB)
Luxembourg	• Department of Health<br>• Red Cross
New Zealand	• Ministry of Health<br>• Ministry for the Environment<br>• Ministry of Civil Defence and Emergency Management<br>• Institute of Environmental Science and Research Limited
Switzerland	• Office fédéral de la santé publique (OFSP)<br>• L'Office fédérale de l'environnement (OFEV)<br>• Organe consultatif sur les changements climatiques (OcCC)
United Kingdom	• Public Health England (PHE)<br>• Environment Agency (EA)<br>• Department of Health (DH)<br>• National Health Service (NHS)<br>• Department for Environment, Food and Rural Affairs<br>• Health Protection Agency<br>• Sustainable Development Unit<br>• Directors of Public Health<br>• Committee on Climate Change<br>• Natural Hazards Partnership (comprised of: Met Office, Environment Agency, Flood Forecasting Centre, Ordenance Survey, Department for Environment Food and Rural Affairs (Defra), Cabinet Office, British Geological Survey, National Centre for Atmospheric Science, National Oceanographic Centre, Centre for Ecology and Hydrology, UK Space Agency, Government Office for Science and the HPA)
United States	• Centers for Disease Control and Prevention (CDC)<br>• Department of Health & Human Services<br>• National Institute of Health (NIH)<br>• Environmental Protection Agency (EPA)<br>• Department of Veteran Affairs (VA)<br>• National Oceanic and Atmospheric Administration (NOAA)<br>• Regional Health Administrator (RHA)<br>• Veterans Health Administration (VHA)

	• Division of the Civilian Volunteer Medical Reserve Corps (DCVMRC) in the Office of the U.S. Surgeon General<br>• Emergency System for Advance Registration of Volunteer Health Professionals (ESAR-VHP)<br>• Department of Homeland Security<br>• Interagency Climate Change Adaptation Task Force<br>• National Association of City and County Health Officials (NACCHO)<br>• Association of State and Territorial Health Officials (ASTHO)<br>• Office of Minority Health (OMH)<br>• Office on Women's Health<br>• Substance Abuse and Mental Health Services Administration (SAMHSA)<br>• U.S. Forest Service

Note: For some health adaptation initiatives reviewed, the responsible department, agency or other body was not specified.

Appendix F: Adaptation and Health System Profiles of Sampled Countries

Table S6. Background information on included countries' adaptation planning and health system.

Country	Adaptation and Health System Information
Australia	Following the Council of Australian Governments (CoAG) request for a national adaptation framework in its Plan of Collaborative Action on Climate Change [4], the Department of Climate Change and Energy Efficiency prepared the country's National Climate Change Adaptation Framework in 2007 [5]. This framework was designed to outline a future agenda for collaboration between governments to support decision-makers [5]. The framework's two priority areas for potential action are: (1) Building understanding and adaptive capacity; and (2) Reducing vulnerability in key sectors and regions [5]. The framework states that a follow-up implementation plan would be developed in 2007, and that the Australian Health Ministers' Conference would develop and implement a national action plan on climate change and health by 2010 [5], however neither of these two documents were ultimately developed. The Australian Government did, however, release a climate change adaptation position paper in 2010, which sets out the Government's vision for adaptation and proposes practical steps [6]. The position paper outlines the national government role in adaptation as: maintaining a strong, flexible economy and a social safety net; leading national reform; managing Commonwealth assets and programs; and national science and information [6]. Though human health is not identified as one of the initial national priorities for adaptation action, it is a priority research theme for the National Climate Change Adaptation Research Facility [6].

	Australia has a federal, decentralized and coordinated state structure [7,8]. The sub-national states have the primary responsibility for public health and the delivery of population health services, and receive funding from the national government [9,10]. Accordingly, the states have a stronger influence on the local level than the national government [9].
Belgium	Coordination in multi-level governance is a key aspect of Belgium's climate change adaptation planning. The *Commission Nationale de Climat* (CNC) [National Climate Commission] was created as part of Article 3 of the 2003 Climate Cooperation Agreement to implement and monitor the Agreement, to harmonize the climate policies developed by the federal and regional governments and to create synergies between them [11]. The CNC is comprised of representatives from the federal and regional governments [12]. Under the instruction of the CNC, the federal and regional governments prepared the National Climate Change Adaptation Strategy together in 2010 [12]. In 2013 the federal government published a draft *Projet de Plan Fédéral 'Adaptation aux changements climatiques'* [Federal Plan Project 'Climate change adaptation'] [13], which was subsequently improved by a public consultation held in 2014 [14]. The Flemish Region published a draft Regional Plan for Adaptation to Climate Change in 2013, and the Walloon and Brussels regions are expected to finalize their plans soon [15]. Together, the finalized federal and regional government climate change adaptation plans will form the basis for a future National Adaptation Plan [15].<br>As a formerly centralized government that began the process of federalism over four decades ago, Belgium has a complex and unique system [16]. At the sub-national level, policy authority has been granted to three territorial authorities (regions) and to three linguistic communities [16]. Social security and health care have been contentious issues in debates of policy devolution in Belgium [16]. Decision-making and implementation of health care policy is fragmented among the federal and sub-national governments [17]. Health policy largely remains under the authority of the federal government, as part of its social security responsibility, but it is the communities that are responsible for public health [16]. At the federal level, the Environment Department of the Federal Public Service (FPS) Health, Food Chain Safety and Environment is responsible for matters concerning climate change [17].
Canada	The Federal Adaptation Policy Framework serves as Canada's adaptation strategy, and outlines the federal role in climate change adaptation: mainstreaming adaptation into policy, generating and sharing knowledge, and capacity building [18]. In 2005, the Canadian Intergovernmental Climate Change Impacts and Adaptation Working Group released the National Climate Change Adaptation Framework, a collaboration between federal and provincial departments and ministries, but the framework was not approved by the federal government and never followed up on [19,20]. Natural Resources Canada has published three vulnerability assessments since 2004 [21-23], alternating between sectoral and regional approaches, and Health Canada has also published a vulnerability assessment focussing on the health sector [24]. The Clean Air Agenda was developed in 2009 as part of the national government's efforts to tackle

	climate change and air pollution. Though otherwise focussed on mitigation, the adaptation theme of the Clean Air Agenda includes several adaptation programs led by Aboriginal Affairs and Northern Development Canada (AANDC), Natural Resources Canada, Health Canada, Environment Canada and the Public Health Agency of Canada.<br>Canada has a federal, decentralized system where health is primarily the responsibility of the 10 provinces and three territories [25]. Each province and territory has their own legislation and regulations for the governance of administration of health care, although the federal government does have some 'steering' responsibilities under the Canada Health Act [25]. Under this decentralized system, Canada's national-level approach to health adaptation has been to prepare vulnerability assessments and conduct research, then allow sub-national governments to use this information as they see fit.
France	Adaptation first emerged in France with the founding of the *Observatoire national sur les effets du réchauffement climatique* [National observatory on the effects of global warming] (ONERC) in 2001, followed by the adoption of a national adaptation strategy in 2006 [26]. France was one of the first European countries to develop a national adaptation strategy (along with Spain and Finland) [27]. In 2011 France adopted a national climate change adaptation plan [26], which outlines five overarching objectives guiding French health adaptation to climate change:<br>• Strengthen climate-health research<br>• Establish or strengthen surveillance of risk factors that can be influenced by climatic conditions (extreme events)<br>• Assess the risk of consequences to human health related to extreme events and appraise the health impacts of adaptation measures, particularly through the creation of the health and climate monitoring group<br>• Develop preventative health measures taking into account the consequences of extreme events and adapt vigilance and warning systems<br>• Raise awareness and educate all stakeholders through targeted training, information and communication<br>Prior to the development of the national climate change adaptation plan, the *Agence française de sécurité sanitaire des aliments (Afssa)* [French food safety agency] also prepared a report on the evaluation of the risk of occurrence and development of animal diseases considering possible global warming [28]. Health is one of several priority sectors for adaptation in France, in part because of its crosscutting nature and implications for other sectors.<br>France has a unitary, coordinated, formally centralized government where health is primarily centralized to the national government [8,29]. Moreover, the 2004 Public Health Act firmly established the

	responsibility of the central state in public health matters, but also emphasized the role of the regional level for organizational issues [29].
Ireland	Ireland's National Climate Change Adaptation Framework, adopted in 2012, guides the country's adaptation response, led by the Department of the Environment, Community and Local Government [30]. The Framework directs local authorities and key climate-sensitive sectors (identified as water, marine, agriculture, forestry, biodiversity, energy, transport, communications, insurance, heritage and health) to develop draft adaptation plans, to be published by mid-2014 [30]. The health sector has yet to publish its draft adaptation plan, but the Department of Health's Statement of Strategy 2015–2017 highlights a health sector climate change adaptation plan as a deliverable for that period [31].<br>The Department of the Environment, Community and Local Government leads and coordinates national adaptation policy to ensure coordination across scales and sectors, particularly for issues that cut across sectors [30]. Ireland describes its "multi-level governance approach" to adaptation as involving (among other aspects): preparing periodic reports on adaptation and anticipated long-range adaptation costs and avoided damages; engagement with key stakeholders to explore impacts and responses; procedures and capacity to undertake emergency response measures when extreme weather events occur; and continued research on climate change impacts [30]. Beyond these examples, the national government does not identify more explicitly how it will put into action the coordination across scales and sectors it describes.<br>Ireland has a unitary, centralized government and a national health insurance health care system [7,32]. In Ireland's health care system, the central government administers the provision of services and regulation, though this responsibility was previously held by decentralized regional health boards until a reform implemented in 2005 [33]. Public health services are also now centralized to the national government [33].
Luxembourg	Luxembourg adopted a National Adaptation Strategy in 2011 which prioritizes biodiversity, water, agriculture and forestry [34]. The strategy does not address the health sector, but Luxembourg's Sixth National Communication to the UNFCCC discusses plans to include additional sectors, including health, to the strategy in the future [34].<br>Luxembourg has a unitary, centralized democracy [7]. The health care system is similarly centralized with little decentralization to the sub-national level [35].
New Zealand	In New Zealand statutory responsibility for consideration of climate change is devolved to local governments [36]. Under the Resource Management Act Section 7(i) amended in 2004, local governments are required to have particular regard for the effects of climate change [37]. Under this institutional arrangement, the central government sets frameworks but devolves powers and functions to sub-national governments [36]. The central New Zealand government has developed adaptation guidance documents for local governments [38], but has not published any national adaptation planning documents.

	Though statutory responsibility for adaptation has been devolved to sub-national governments, New Zealand has the most centralized framework for public health, next to Singapore [10]. New Zealand's health system has undergone many reforms in recent decades, moving toward increased centralization [39]. The national government has more direct control over the health system and health services than most other high-income countries due to its concentrated constitutional power, its dominance over health funding, and in its role as the main provider of hospital services. Due to the lack of literature, it is unclear how the public health risks of climate change will be addressed given the conflicting mandates for local adaptation and national health planning.
Switzerland	In 2011 the Swiss federal government implemented the *loi sur le CO_2* [CO_2 Act] (updated in 2013) which includes an article requiring coordination of adaptation measures [40]. The federal government (hereafter the Confederation) released its first adaptation strategy in 2012 [41], which was then further developed into an action plan in 2014 [42]. The Federal Office for the Environment (OFEV) is responsible for the overall development and cross-sectoral coordination of the national adaptation strategy and action plan, as well as vertical coordination with sub-national governments. It shares responsibility with the Federal Office of Public Health (OFSP) for health sector adaptation measures [41,42].<br>Switzerland is a federal, decentralized democracy, with 26 sub-national cantons [7]. The cantons have the most responsibility for the health system, and each canton has its own health policies and laws [43]. The Swiss health system is fragmented and thus lacks a consistent national health policy [43].<br>Adaptation to climate change in the health sector appears to have more mechanisms for coordination than the characterization of the Swiss health system overall. The Swiss adaptation action plan clearly lays out that the Confederation will work with sub-national governments and which sectors will work together on cross-sectoral issues [42]. The Confederation plans to support the cantons, municipalities and cities already working actively on adaptation by supplying targeted information. For instance, the Confederation prepared a climate change adaptation guidebook for Swiss municipalities, including consideration of health [44]. One of the Confederation's objectives is to collaborate by ensuring transfer of knowledge between levels and also to coordinate adaptation strategies and measures to ensure the needs of the cantons, cities and municipalities are included in the implementation and development of the national adaptation strategy, while ensuring that adaptation in Switzerland is performed consistently and efficiently. The cantons must submit reports every four to six years (beginning in 2015) to the OFEV detailing their planned or implemented adaptation measures. The OFEV will compile the results as a basis for coordination and consider them for further developing the national adaptation strategy [42].
United Kingdom	The United Kingdom was among the first European countries to develop a national adaptation strategy [27]. The Climate Change Act 2008 set out a legislative framework for adaptation, requiring preparation of a National Adaptation Programme and undertaking of regular risk assessments by the government [45,46].

	Despite this early legislation and planning work, the earliest health adaptation initiatives identified online are from two 2012 documents and comprise a climate change risk assessment, an assessment of the health effects of climate change in the UK, the creation of the Extreme Events and Health Protection Section (EEHPS) and the creation of the daily hazard assessment (early warning system) [47,48]. The health vulnerability assessment references a 2002 health vulnerability assessment [48], but the 2002 document is no longer available online, and was thus not included in this study.<br>Most health adaptation initiatives in the United Kingdom are implemented by Department of Health or other agencies that support it, including Public Health England (PHE) and the National Health Service (NHS). In a self-assessment of adaptive capacity, the Department of Health scored itself 7/25 against five levels of progress for each of five themes (leadership, policy and strategy, people, partnerships and processes) [45]. The Committee on Climate Change describes this score as "relatively low considering the significant risk that extreme weather events pose to public health and well-being"[45]. Most health adaptation initiatives are implemented or planned by partnerships between multiple agencies or departments, including several initiatives that include multiple sectors.<br>Health planning and policy is developed by the central government in the United Kingdom, but there is some decentralization in the implementation of the health system [49]. In a sample of 28 urban areas, 75% were found to consider health aspects in climate change adaptation measures [50]. From 2008 to 2011 the Department for Communities and Local Government implemented the National Indicator Set (NIS), which includes National Indicator (NI) 188: "Planning to Adapt to Climate Change." The rationale behind NI 188 was to "ensure local authority preparedness to manage risks to service delivery, the public, local communities, local infrastructure, businesses and the natural environment from a changing climate, and to make the most of new opportunities"[51]. Though the NIS was abolished in 2011, NI 188 demonstrates one planning instrument a centralized unitary government can use to promote, facilitate and influence adaptation among local authorities [51].
United States	In the last year, public health adaptation to climate change in the United States has made significant progress, driven in part by the President's executive order for each federal agency to prepare for climate change. Executive order 13514 "Federal Leadership in Environmental, Energy and Economic Performance" (October 2009) requires each federal agency to develop, implement and annually update an integrated Strategic Sustainability Performance Plan including an evaluation of the risks posed by climate change and the development of a climate change adaptation plan. The Department of Health and Human Services (DHHS) has subsequently developed a draft climate change adaptation plan and a finalized, comprehensive plan [52,53]. The DHHS adaptation plan is detailed, involves many departments and agencies (including the Department of Veteran Affairs, the National Association of City and County Health Officials (NACCHO) and the Association of State and Territorial Health Officials (ASTHO)), is part of a larger coordinated effort on

	adaptation (The Interagency Climate Change Adaptation Task Force was developed as part of the Executive Order), and places the DHHS in a leadership role in public health adaptation to climate change in the United States. Based on publically available information, 18 departments, agencies, divisions or offices report implementing or planning health adaptation initiatives, most falling under the jurisdiction of the DHHS. Typically many federal public health programs have a crisis orientation not suited to longer term issues [10], thus the DHHS's comprehensive climate change adaptation plan is a shift from the norm. The United States is a federal, decentralized democracy where states have explicit authority to protect their residents' health under the US constitution [8,10]. The federal government also has a responsibility to promote public health, but ultimately it must rely on the states for compliance and implementation of federal guidelines [10]. Following the implementation of the Executive Order 13514 and the development of the Interagency Climate Change Adaptation Task Force, the federal government is taking a larger role in national planning and leadership [54]. Currently only 10 out of 50 American states have detailed health sector specific actions recommended, and those are predominantly coastal, democratic states [54]. To date, the federal government has not guided or coordinated state adaptation, and most of the leadership and resources for state adaptation has come from the state level [55].

References

1. Austin, S.E.; Ford, J.D.; Berrang-Ford, L.; Araos, M.; Parker, S.; Fleury, M.D. Public Health Adaptation to Climate Change in Canadian Jurisdictions. *Int. J. Environ. Res. Public Health* **2015**, *12*, 623–651.
2. Panic, M.; Ford, J. A Review of National-Level Adaptation Planning with Regards to the Risks Posed by Climate Change on Infectious Diseases in 14 OECD Nations. *Int. J. Environ. Res. Public Health* **2013**, *10*, 7083–7109.
3. Biagini, B.; Bierbaum, R.; Stults, M.; Dobardzic, S.; McNeeley, S.M. A typology of adaptation actions: A global look at climate adaptation actions financed through the Global Environment Facility. *Glob. Environ. Chang.* **2014**, *25*, 97–108.
4. Council of Australian Governments. *Council of Australian Governments' Plan for Collaborative Action on Climate Change*; Council of Australian Governments: Canberra, Australia, 2006.
5. Department of Climate Change and Energy Efficiency. *National Climate Change Adaptation Framework*; Department of Climate Change and Energy Efficiency: Canberra, Australia, 2007.
6. Australian Government. *Adapting to Climate Change in Australia—An Australian Government Position Paper*; Department of Climate Change, Ed.; Australian Government: Canberra, Australia, 2010.
7. Lijphart, A. *Patterns of Democracy: Government Forms and Performance in Thirty-Six Countries*; Yale University Press: New Haven, CT, USA, 2012.
8. Pollitt, C.; Bouckaert, G. *Public Management Reform: A Comparative Analysis-New Public Management, Governance, and the Neo-Weberian State*; Oxford University Press: Oxford, UK, 2011.
9. Healy, J.; Sherman, E.; Lokuge, B. *Australia: Health System Review*; European Observatory on Health Systems and Policies: Copenhagen, Denmark, 2006; pp. 1–158.
10. Blank, R.H.; Burau, V. *Comparative Health Policy*, 4 ed.; Palgrave Macmillan: London, United Kingdom, 2014.
11. Sénat de Belgique. Projet de loi portant assentiment à l'Accord de coopération entre l'État fédéral, la Région flamande, la Région wallonne et la Région de Bruxelles-Capitale relatif à l'établissement, l'exécution et le suivi d'un Plan National Climat, ainsi que l'établissement de rapports, dans le cadre de la Convention-cadre des Nations unies sur les Changements climatiques et du Protocole de Kyoto, conclu à Bruxelles le 14 novembre 2002. In *Doc 50 2376/001*; Belgique, Chambre des Représentants de Belgique: Bruxelles, Belgium, 2003.
12. Commission Nationale Climat. *Belgian National Climate Change Aadptation Strategy*; Flemish Environment Nature and Energy Department: Brussels, Belgium, 2010.
13. Service Public Fédéral Belge. *Projet de Plan Fédéral 'Adaptation aux Changements Climatiques'*; 13. Service Public Fédéral Belge: Brussels, Belgium, 2013.
14. Service Fédéral. Plan Fédéral Adaptation. Available online: http://www.climat.be/fr-be/politiques/politique-belge/politique-federale/plan-federal-adaptation/ (accessed on 18 June 2015).
15. Commission Nationale Climat. *Belgium's Sixth National Communication: Under the United Nations Framework Convention on Climate Change*; Federal Public Service Health, Food Chain Safety and Environment: Brussels, Belgium, 2013.
16. Laible, J. Devolution, Nationalism, and the Limits of Social Solidarity: The Federalization of Health Policy in Belgium. In *Federalism and Decentralization in European Health and Social Care*; Costa-Font, J., Greer, S.L., Eds.; Palgrave Macmillan: London, UK, 2012; p. 228.
17. Gerkins, S.; Merkur, S. *Belgium: Health System Review*; European Observatory on Health Systems and Policies: Copenhagen, Denmark, 2010; pp. 1–266.
18. Government of Canada. *Federal Adaptation Policy Framework*; Environment Canada, Ed.; Government of Canada: Gatineau, QC, Canada, 2011.
19. Brooks, M.; Gagnon-Lebrun, F.; Harvey, H.; Sauvé, C.; ÉcoRessources Consultants. *Prioritizing Climate Change Risks and Actions on Adaptation: A Review of Selected Institutions, Tools and Approaches: Final Report*; 1100115986; Policy Research Initiative, Government of Canada: Ottawa, ON, Canada, 2009.
20. Westerhoff, L.; Keskitalo, E.C.H.; McKay, H.; Wolf, J.; Ellison, D.; Botetzagias, I.; Reysset, B. Planned adaptation measures in industrialised countries: A comparison of select countries within and outside the EU. In *Developing Adaptation Policy and Practice in Europe: Multi-Level Governance of Climate Change*; Keskitalo, E.C.H., Ed.; Springer: Dordrecht, The Netherlands, 2010; pp. 271–338.
21. Lemmen, D.S.; Warren, F.J.; Lacroix, J.; Bush, E. *From Impacts to Adaptation: Canada in a Changing Climate 2007*; Natural Resources Canada, Government of Canada: Ottawa, ON, Canada, 2008.

22. Warren, F.J.; Lemmen, D.S. *Canada in a Changing Climate: Sector Perspectives on Impacts and Adaptation*; Government of Canada: Ottawa, ON, Canada, 2014; p. 286.
23. Lemmen, D.S.; Warren, F.J. *Climate Change Impacts and Adaptation: A Canadian Perspective*; Natural Resources Canada: Ottawa, ON, Canada, 2004.
24. Séguin, J. *Human Health in a Changing Climate: A Canadian Assessment of Vulnerabilities and Adaptive Capacity*; Health Canada: Ottawa, ON, Canada, 2008.
25. Marchildon, G.P. *Canada: Health System Review*; European Observatory on Health Systems and Policies: Copenhagen, Denmark, 2013.
26. Ministère de l'Écologie du Développement durable et de l'Énergie. *Sixième Communication Nationale de la France à la Convention-Cadre des Nations unies sur les Changements Climatiques*; Ministère de l'Écologie du Développement durable et de l'Énergie: Paris, France, 2013.
27. European Environment Agency. *National Adaptation Policy Processes in European Countries—2014*; European Environment Agency: Copenhagen, Denmark, 2014.
28. Rodhain, F.; Albina, E.; André-Fontaine, G.; Armengaud, M.; Dreyfuss, G.; Dufour, B.; Duvallet, G.; Gauchard, F.; Moutou, F.; Zientara, S. *Rapport sur l'évaluation du risque d'apparition et de développement des maladies animales compte tenu d'un éventuel réchauffement climatique*; AFSSA: Maisons-Alfort, France, 2005.
29. Chevreul, K.; Durand-Zaleski, I.; Bahrami, S.; Hernández-Quevedo, C.; Mladovsky, P. *France: Health System Review*; European Observatory on Health Systems and Policies: Copenhagen, Denmark 2010; pp. 1–291.
30. Department of the Environment Community and Local Government. National Climate Change Adaptation Framework. 2012.
31. Department of Health. *Department of Health Statement of Strategy 2015–2017*; Department of Health: Dublin, Ireland, 2014.
32. Böhm, K.; Schmid, A.; Götze, R.; Landwehr, C.; Rothgang, H. *Classifying OECD Healthcare Systems: A Deductive Approach*; University of Bremen: Bremen, Germany, 2012; TranState Working Papers, No. 65.
33. McDaid, D.; Wiley, M.; Maresso, A.; Mossialos, E. *Ireland: Health System Review*; European Observatory on Health Systems and Policies: Copenhagen, Denmark, 2009; pp. 1–268.
34. Ministry of Sustainable Development and Infrastructure. *Sixth National Communication of Luxembourg under the United Nations Framework Convention on Climate Change: including Luxembourg's Biennial Report No 1*; Ministry of Sustainable Development and Infrastructure: Luxembourg, Luxembourg, 2013.
35. European Observatory on Health Care Systems. *Health Care Systems in Transition: Luxembourg*; European Observatory on Health Care Systems: Copenhagen, Denmark, 1999.
36. Lawrence, J.; Sullivan, F.; Lash, A.; Ide, G.; Cameron, C.; McGlinchey, L. Adapting to changing climate risk by local government in New Zealand: institutional practice barriers and enablers. *Local Environ.* **2013**, *20*, 298–320.
37. Reisinger, A.; Wratt, D.; Allan, S.; Larsen, H. The role of local government in adapting to climate change: lessons from New Zealand. In *Climate Change Adaptation in Developed Nations*; Ford, J., Berrang-Ford, L., Eds.; Springer: Dordrecht, The Netherlands, 2011; pp. 303–319.
38. Ministry for the Environment. *Climate Change Effects and Impacts Assessment: A Guidance Manual for Local Government in New Zealand*, 2nd ed.; Ministry for the Environment: Wellington, New Zealand.
39. Ashton, T.; Tenbensel, T. Reform and Re-reform of the New Zealand System. In *Seven Countries, Seven Reform Models : The Healthcare Reform Experience of Israel, the Netherlands, New Zealand, Singapore, Switzerland and Taiwan—Healthcare Reforms under the Radar Screen*; Okma, K.G.H., Crivelli, L., Klein, R., Eds.; World Scientific Publishing Co.: Singapore, 2009.
40. L'Assemblée Fédérale de la Confédération Suisse. Loi Fédérale sur la Réduction des Émissions de CO_2 (Loi sur le CO_2). In *641.71*; L'Assemblée Fédérale de la Confédération Suisse: Bern, Switzerland, 2013.
41. Swiss Confederation. *Adaptation aux Changements Climatiques en Suisse: Objectifs, défis et Champs D'action—Premier volet de la Stratégie du Conseil fédéral du 2 mars 2012*; Swiss Confederation: Bern, Switzerland, 2012.
42. Swiss Confederation. *Adaptation aux Changements Climatiques en Suisse: Plan D'action 2014–2019—Deuxième volet de la stratégie du Conseil Fédéral*; Swiss Confederation: Bern, Switzerland, 2014.
43. Gerritzen, B.C.; Kirchgässner, G. Federalism in health and social care in Switzerland. In *Federalism and decentralization in European Health and Social Care*; Costa-Font, J., Greer, S.L., Eds.; Palgrave Macmillan: London, United Kingdom, 2013; pp. 250–271.
44. Office Fédérale de l'Environnement. *Adaptation aux Changements Climatiques dans les Villes Suisses [Adaptation to Climatic Changes in Swiss Cities]*; Section Climat: Bern, Switzerland, 2012.

45. Committee on Climate Change. *How Well Prepared Is the UK for Climate Change? (Adaptation Sub-Committee Progress Report 2010)*; Committee on Climate Change: London, UK, 2010.
46. Ranger, N.; Millner, A.; Dietz, S.; Fankhauser, S.; Lopez, A.; Ruta, G. *Adaptation in the UK: A Decision-Making Process*; Environment Agency: Rotherham, UK, 2010.
47. HM Government. *Climate Change Risk Assessment: Government Report*; Department for Environment, Food and Rural Affairs: London, UK, 2012.
48. Health Protection Agency. *Health Effects of Climate Change in the UK 2012: Current Evidence, Recommendations and Research Gaps*; Health Protection Agency: Chilton, UK, 2012.
49. Boyle, S. *United Kingdom (England): Health System Review*; European Observatory on Health Systems and Policy: Copenhagen, Denmark, 2011.
50. Heidrich, O.; Dawson, R.J.; Reckien, D.; Walsh, C.L. Assessment of the climate preparedness of 30 urban areas in the UK. *Clim. Chang.* **2013**, *120*, 771–784.
51. Audit Commission. *National Indicator for Local Authorities and Local Authority Partnerships: Local Economy and Environmental Sustainability NIS Definitions*; Community and Local Governament: London, UK, 2008.
52. Department of Health & Human Services. *HHS Climate Adaptation Plan*; Department of Health & Human Services: Washington, DC, USA, 2014.
53. Department of Health & Human Services. *Draft HHS Climate Change Adaptation Plan*; Department of Health & Human Services: Washington, DC, USA, 2012.
54. Lysák, M.; Bugge-Henriksen, C. Current status of climate change adaptation plans across the United States. *Mitig. Adapt. Strateg. Glob. Chang.* **2014**, doi:10.1007/s11027-014-9601-4.
55. Moser, S.C. Entering the period of consequences: the explosive US awakening to the need for adaptation. In *Climate Change Adaptation in Developed Nations*; Ford, J.D., Berrang-Ford, L., Eds.; Springer: Dordrecht, The Netherlands, 2011; pp. 33–49.

© 2016 by the authors. Submitted for possible open access publication under the terms and conditions of the Creative Commons Attribution (CC-BY) license (http://creativecommons.org/licenses/by/4.0/).
